# Five hTRPA1 Agonists Found in Indigenous Korean Mint, *Agastache rugosa*

**DOI:** 10.1371/journal.pone.0127060

**Published:** 2015-05-15

**Authors:** Hana Moon, Min Jung Kim, Hee Jin Son, Hae-Jin Kweon, Jung Tae Kim, Yiseul Kim, Jaewon Shim, Byung-Chang Suh, Mee-Ra Rhyu

**Affiliations:** 1 Research Group of Food Functionality, Korea Food Research Institute, Bundang-gu, Sungnam-si, Gyeonggi-do, Republic of Korea; 2 Department of Brain Science, Daegu Gyeongbuk Institute of Science and Technology (DGIST), Daegu, Republic of Korea; Duke University Medical Center, UNITED STATES

## Abstract

Transient receptor potential ankyrin1 (TRPA1) and transient receptor potential vanilloid 1 (TRPV1) are members of the TRP superfamily of structurally related, nonselective cation channels and mediators of several signaling pathways. Previously, we identified methyl syringate as an hTRPA1 agonist with efficacy against gastric emptying. The aim of this study was to find hTRPA1 and/or hTRPV1 activators in *Agastache rugosa* (*Fisch*. *et Meyer*) *O*. *Kuntze* (*A*.*rugosa*), commonly known as Korean mint to improve hTRPA1-related phenomena. An extract of the stem and leaves of *A*.*rugosa* (Labiatae) selectively activated hTRPA1 and hTRPV1. We next investigated the effects of commercially available compounds found in *A*.*rugosa* (acacetin, 4-allylanisole, *p*-anisaldehyde, apigenin 7-glucoside, L-carveol, β-caryophyllene, *trans*-*p*-methoxycinnamaldehyde, methyl eugenol, pachypodol, and rosmarinic acid) on cultured hTRPA1- and hTRPV1-expressing cells. Of the ten compounds, *L*-carveol, *trans*-*p*-methoxycinnamaldehyde, methyl eugenol, 4-allylanisole, and *p*-anisaldehyde selectively activated hTRPA1, with EC50 values of 189.1±26.8, 29.8±14.9, 160.2±21.9, 1535±315.7, and 546.5±73.0 μM, respectively. The activities of these compounds were effectively inhibited by the hTRPA1 antagonists, ruthenium red and HC-030031. Although the five active compounds showed weaker calcium responses than allyl isothiocyanate (EC_50_=7.2±1.4 μM), our results suggest that these compounds from the stem and leaves of *A*.*rugosa* are specific and selective agonists of hTRPA1.

## Introduction

Transient receptor potential ankyrin1 (TRPA1), a member of the large TRP family of ion channels, is widely expressed in peripheral and sensory neurons [1–3]. In addition, many cell types, tissues, and organs including enterochromaffin cells, airway epithelial cells, the brain, and hair cells, express TRPA1. TRPA1 is involved in diverse activities, including acute and chronic pain and inflammation, delayed gastric emptying, cold sensation (<17°C), and chemosensation [4,5]. Many of the oxidants produced during inflammatory reactions, including nitro-oleic acid, 4-hydroxynonenal, and hydrogen peroxide, are TRPA1 agonists [6,7]. Transient receptor potential vanilloid 1 (TRPV1), another member of the TRP superfamily, is partially co-expressed with TRPA1 in sensory nerve endings and has been linked to peripheral inflammation, heat sensation (43–52°C), and neuronal damage [8,9]. Therefore, the discovery of new compounds targeting TRPA1 and/or TRPV1 could contribute to various functions involved in TRPA1 and/or TRPV1.

A large number of pungent TRPA1 agonists have been discovered in foods. A variety of isothiocyanate compounds, including allyl isothiocyanate (AITC) in wasabi, benzyl isothiocyanate in yellow mustard, phenylethyl isothiocyanate in Brussels sprouts, isopropyl isothiocyanate in nasturtium seeds, methyl isothiocyanate in capers [10], allicin in garlic, and cinnamaldehyde (CALD) in cinnamon oil, have been identified as strong activators of TRPA1 [11]. Non-pungent compounds such as capsiate [12] and the fatty acids in royal jelly are also TRPA1 activators [13]. Allicin in garlic activates both TRPA1 and TRPV1. Foods such as hot pepper, black pepper, garlic, ginger, and sanshō contain TRPV1-activating compounds such as capsaicin and gingerol.

Culinary plants in Korea also contain TRPA1-and TRPV1-activating compounds. In previous studies, we identified methyl syringate in the first leaves of *Kalopanax pictus* Nakai (Araliaceae) as a TRPA1 agonist [14] that delays gastric emptying [15]. Here, we demonstrate that the stem and leaves of *Agastache rugosa (Fisch*. *et Meyer) O*. *Kuntze* (*A*.*rugosa*), commonly known as Korean mint, a member of the mint family (Labiatae), contains TRPA1- or TRPV1-active compounds. *A*.*rugosa* is indigenous Korean mint, but also distributed in China, Japan, and Siberia. In Korea, the sprouts and shoots of *A*.*rugosa* are used as foods and the aerial parts have been used as medicine. Traditionally, *A*. *rugosa* has been used for the treatment of cholera, vomiting, and miasma. It has been reported to have anti-tumor, anti-fungal, anti-atherogenic, and anti-inflammatory activities [16–18]. Considering that TRPA1 and TRPV1 are involved in anti-inflammatory effects, TRPA1 or TRPV1 active compounds can exist in *A*.*rugosa*, and those compounds may affect TRPA1 or TRPV1-related functions.

We investigated the effects of the stem and leaves of *A*.*rugosa* on hTRPV1 and hTRPA1 and identified specific chemical compounds in the stem and leaves of *A*.*rugosa* that activate hTRPA1 or hTRPV1. Ten commercially available chemicals in *A*.*rugosa* (acacetin, 4-allylanisole, *p*-anisaldehyde, apigenin 7-glucoside, *L*-carveol, *β*-caryophyllene, *trans*-*p*-methoxycinnamaldehyde, methyl eugenol, pachypodol, and rosmarinic acid) were selected based on Dr. Duke's Phytochemical and Ethnobotanical Database [19–21]. We determined the efficacies of *A*.*rugosa* and each compound for hTRPA1 and hTRPV1 by monitoring the changes in cytosolic Ca^2+^ influx in hTRPA1- and hTRPV1-expressing cells using the fluorescent dyes, Fura-2 AM and Fluo-4 AM.

## Materials and Methods

### Materials

**Fig 1:**
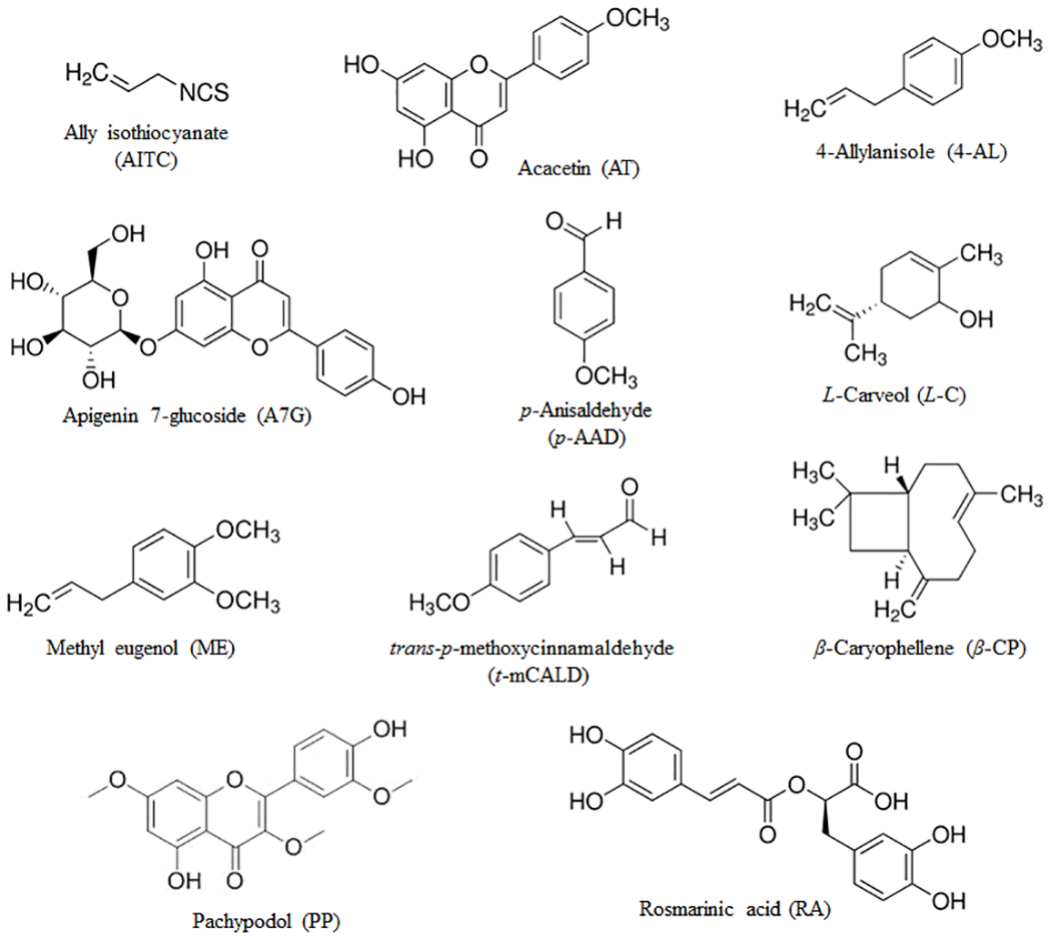
Chemical structure of AITC and the ten compounds from *A*.*rugosa*.

AITC, capsaicin, ruthenium red (RR), HC-030031, capsazepine (CPZ), acacetin, 4-allylanisole, *p*-anisaldehyde, apigenin 7-glucoside, *L*-carveol, *β*-caryophyllene, *trans*-*p*-methoxycinnamaldehyde, methyl eugenol, rosmarinic acid, and dimethyl sulfoxide (DMSO) were purchased from Sigma-Aldrich (St. Louis, MO, USA). Pachypodol was obtained from Chem Faces (Hubei, China). The media used for cell culture were obtained from Life Technologies, Inc. (Grand Island, NY, USA). The structures of AITC, acacetin, 4-allylanisole, *p*-anisaldehyde, apigenin 7-glucoside, *L*-carveol, *β*-caryophyllene, *trans*-*p-*methoxycinnamaldehyde, methyl eugenol, pachypodol, and rosmarinic acid were shown in Fig 1.

### Plant extract

The stem and leaves of *A*. *rugosa* was obtained from HANTAEK Botanical Garden (365 Oksan-ri, Baegam-myeon, Cheoin-gu, Yongin-si, Gyeonggi-do, Korea). The stem and leaves were freeze-dried and milled with a commercial food mixer. Milled stem and leaves of *A*. *rugosa* were extracted by 80% ethanol using homogenizer and the extract was evaporated under reduced pressure at 37–40°C, lyophilized to a powder, and stored at -80°C until use. The extract was dissolved in DMSO to give 300 mg/ml solutions as a stock solution. The sample was further diluted in assay buffer for the bioassay on the day of the experiment to give final concentration of 300 μg/ml containing 0.1% DMSO. Voucher specimen No. AR001 had been deposited at Korea Food Research Institute, Gyeonggi-do, Korea.

### Cell culture and transfection

Flp-In 293 cells stably expressing hTRPA1 [22] were a gift from Dr. Takumi Misaka (University of Tokyo, Tokyo, Japan). The hTRPA1-expressing cells were maintained in Dulbecco’s modified Eagle’s medium (DMEM; Invitrogen, Carlsbad, CA, USA) containing 10% fetal bovine serum (FBS; Invitrogen) and 0.2% hygromycin B (Invitrogen). Flp-In 293 cells (Invitrogen: R750-07) were maintained in DMEM containing 10% FBS. All cells were incubated at 37°C in a humidified atmosphere containing 5% CO_2_. Cultured hTRPA1-expressing cells and Flp-In 293 cells were seeded onto 96-well black-wall plates for 24 h prior to their use in experiments.

For the transient expression of hTRPA1, HEK293T cells (ATCC: CRL-11268) cultured at 37°C in DMEM supplemented with 10% FBS and 1% penicillin/streptomycin (Invitrogen) were transfected using Lipofectamine 2000 (Invitrogen) and 0.1μg of cDNA encoding tetrameric red fluorescence protein (DsRed) was co-transfected as a marker for successfully transfected cells. The hTRPA1expressing plasmid was generously given to us from Kyeongjin Kang (Sungkyunkwan University School of Medicine, Suwon, Korea). The next day, cells were plated onto poly-L-lysine (0.1 mg/ml, Sigma-Aldrich) coated chips, and the fluorescent cells were studied within 2 days after transfection.

The hTRPV1 used for transient transfection was cloned by OriGene (Rockville, MD, USA; NCBI accession number: NG_029716.1). The hTRPV1 construct was cloned into pEAK10 (Edge Biosystems, Gaithersburg, MD, USA) and the nucleotide sequence of the hTRPV1 gene was confirmed by sequencing with an ABI 3130 DNA genetic analyzer (Applied Biosystems, Foster City,CA, USA). Next, the hTRPV1 expression plasmid was transiently transfected using Lipofectamine 2000. HEK293T cells cultured at 37°C in DMEM supplemented with 10% FBS and 1% penicillin/streptomycin (Invitrogen) were seeded onto 100 mm dishes and transfected with the hTRPV1 expression plasmid using Lipofectamine 2000. After 6 h, the transfected cells were seeded onto 96-well black-wall plates for 24 h prior to their use in experiments.

### Ca^2+^ imaging of the cellular responses of hTRPA1- and hTRPV1-expressing cells

Non-hTRPA1-expressing Flp-In 293 cells, HEK293T cells, hTRPA1-Flp-In 293 stable cells, and HEK293T cells transiently expressing hTRPV1 were seeded onto 96-well black-wall imaging plates (BD Falcon Labware, Franklin Lakes, NJ, USA) for 24 h prior to their use in experiments. After 24 h, the cells were washed with assay buffer (130 mM NaCl, 10 mM glucose, 5 mM KCl, 2 mM CaCl_2_, 1.2 mM MgCl_2_, and 10 mM HEPES [pH 7.4]) and loaded with the Ca^2+^ indicator dye Fura-2AM (5 μM; Invitrogen) in assay buffer for 30 min at 27°C. The cells were rinsed with assay buffer, incubated in 100 μl of assay buffer for >10 min, and then treated with ligand by adding 100 μl of the ligand solution. The ligands were AITC, capsaicin, and ten commercially available compounds found in *A*.*rugosa* (acacetin, 4-allylanisole, *p*-anisaldehyde, apigenin 7-glucoside, *L*-carveol, *β*-caryophyllene, *trans*-*p*-methoxycinnamaldehyde, methyl eugenol, pachypodol, and rosmarinic acid) (Fig 1). AITC and capsaicin, specific agonists of hTRPA1 and hTRPV1, respectively, were used as positive controls. The final concentrations were as follows: AITC, 0.03 mM; acacetin, *trans*-*p*-methoxycinnamaldehyde, and rosmarinic acid, 0.1 mM; *L*-carveol, methyl eugenol, and pachypodol, 0.3 mM; and 4-allylanisole, *p*-anisaldehyde, apigenin 7-glucoside, and *β*-caryophyllene, 1 mM. The fluorescence intensity of Fura-2 AM excited at 340 and 380 nm was measured at 510 nm using a computer-controlled filter changer (Lambda DG4; Sutter Instrument Co., San Rafael, CA, USA), an Andor Luca CCD camera (Andor Technology, Belfast, Northern Ireland), and an inverted fluorescence microscope (IX-71; Olympus, Tokyo, Japan). Images were recorded at 3 s intervals and were analyzed using MetaFluor software (Molecular Devices, Sunnyvale, CA, USA).

### Measurement of the cytosolic Ca^2+^ levels in hTRPA1-expressing cells using a fluorescence plate reader

hTRPA1-Flp-In 293 stable cells were seeded onto 96-well black-wall CellBIND Surface plates (Corning Inc., Corning, NY, USA) 24h before the assay. The cells were loaded with 5 μM Fluo-4AM (Molecular Probes, Eugene, OR, USA) in assay buffer for 30 min at 27°C, washed with assay buffer, and incubated for 15 min at 27°C. Subsequently, the cytosolic Ca^2+^ concentration was measured using a Flex StationIII microplate reader (Molecular Devices). Sample solutions were loaded after a 17s baseline scan and ligand-induced changes in fluorescence intensity (excitation, 486 nm; emission, 516 nm; cutoff, 515 nm) were monitored at 2.1s intervals for 120s. The response of each well is represented as the change in relative fluorescence units (ΔRFU), which was defined as the maximum fluorescence value minus the minimum fluorescence value. All experiments were performed at least three times. Plots of amplitude versus ligand concentration were fitted using Hill’s equations. To investigate inhibition, samples were treated with 30 μM RR or 100 μM HC-030031 in some experiments.

### Whole-cell patch clamp recording

Cells were voltage clamped in the whole-cell recording configuration at room temperature (22–25°C). The resistance of electrodes pulled from glass micropipette capillaries (Sutter Instrument Co., Novato, CA, USA) was 2–2.5 MΩ with >60% compensation of series resistance errors. Fast and slow capacitances were compensated before application of the test pulse. Membrane currents were recorded using a HEKA EPC-10 amplifier with pulse software (HEKA Elektronik, Lambrecht, Germany). The pipette solution used for recording hTRPA1 currents contained 140 mM KCl, 5 mM MgCl_2_, 10 mM HEPES, 0.1 mM 1,2-bis(2-aminophenoxy)ethane-N,N,N',N'-tetraacetic acid (BAPTA), 3 mM Na_2_ATP, and 0.1 mM Na_3_GTP (adjusted to pH 7.4 with KOH). The external Ringer’s solution used for recording hTRPA1 currents contained 160 mM NaCl, 5 mM KCl, 1 mM MgCl_2_, 2 mM EGTA, 10 mM glucose, and 10 mM HEPES (adjusted to pH 7.4with NaOH). hTRPA1 currents were recorded by holding the cell at -70 mV. The following reagents were obtained: BAPTA, Na_2_ATP, Na_3_GTP, and EGTA (Sigma-Alrich), HEPES (Calbiochem, San Diego, CA, USA), and other chemicals (Merck, Township, NJ, USA).

### Statistical analysis

Dose-response analyses were carried out with GraphPad Prism (GraphPad Software Inc., San Diego, CA, USA). The data represent the mean ± SEM. The results were analyzed using a student t-test.

## Results

### Effects of *A*.*rugosa* and its constituents on hTRPA1-and hTRPV1-expressing cells

**Fig 2:**
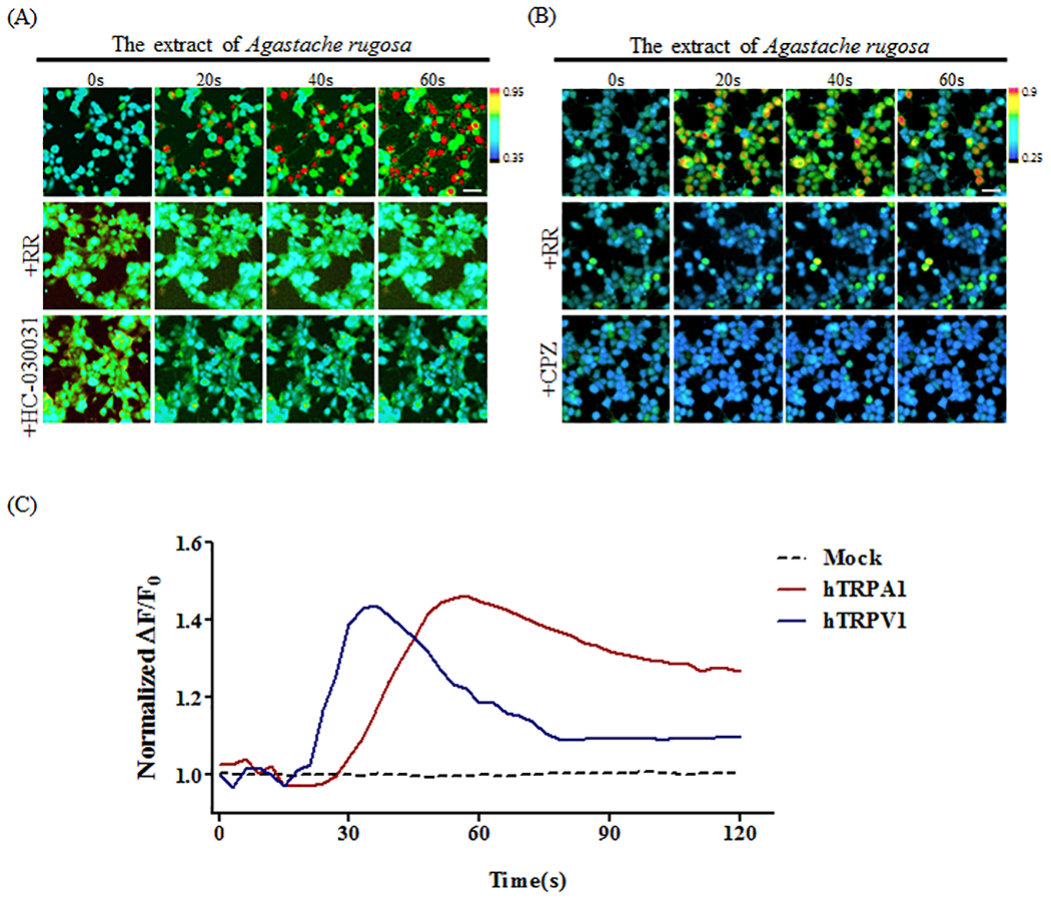
Effect of *A*.*rugosa* on hTRPA1- and hTRPV1-expressing cells. hTRPA1-Flp-In 293 stable cells (A) and HEK293T cells transiently expressing hTRPV1 (B) were loaded with Fura-2 AM and representative ratiometric Ca^2+^ images were obtained at 0, 20, 40, and 60 s after stimulation by the extract of *A*.*rugosa* (300 μg/mL). The specificities of *A*.*rugosa* for hTRPA1 or hTRPV1 were analyzed using antagonists: 30 μM RR and 100 μM HC-030031 for hTRPA1, and 30 μM RR and 5 μM CPZ for hTRPV1. (C) The kinetics of the calcium influx induced by *A*.*rugosa* were quantitatively calculated from Fura-2 ratiometric Ca^2+^ images on HEK293T cells expressing hTRPA1 (red), hTRPV1 (blue), and mock (black).

By Ca^2+^ imaging analysis, an 80% ethanol extract of the stem and leaves of *A*.*rugosa* selectively activated both hTRPA1 and hTRPV1 in a time-dependent manner (Fig 2). The Ca^2+^ responses induced by AITC and the extract were inhibited by the general TRP channel blocker, RR (30 μM) and a selective TRPA1 blocker, HC-030031 (100 μM) in hTRPA1-Flp-In 293 stable cells, and by RR (30 μM) and CPZ (5 μM) in HEK293T cells transiently expressing hTRPV1.

**Fig 3:**
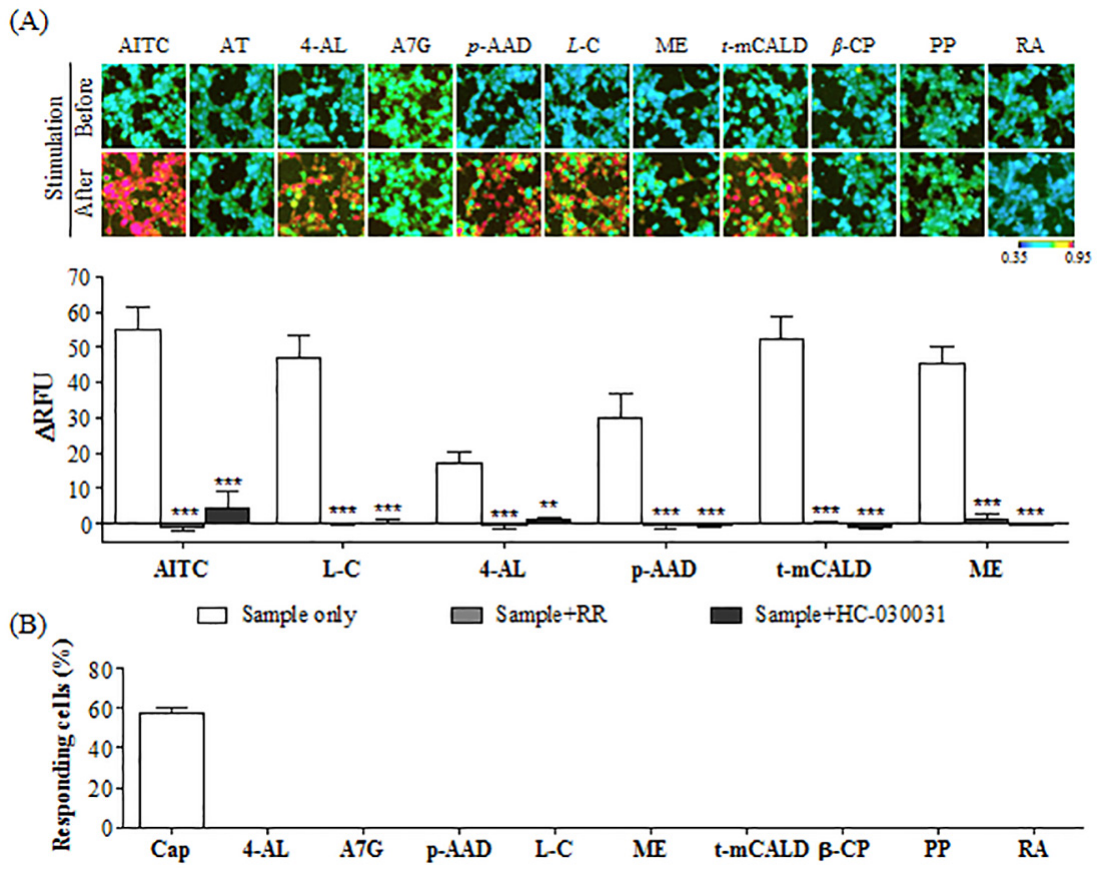
Effects of the ten compounds from *A*.*rugosa* on hTRPA1- and hTRPV1-expressing cells. (A) The activities of the ten compounds from *A*. *rugosa* including acacetin (1 mM), 4-allylanisole (1 mM), apigenin 7-glucoside (1 mM), *p*-anisaldehyde (1 mM), *L*-carveol (0.3 mM), methyl eugenol (0.3 mM), *trans*-*p*-methoxycinnamaldehyde (0.1 mM), *β*-caryophyllene (1 mM), pachypodol (0.3 mM), and rosmarinic acid (0.1 mM) were analyzed in hTRPA1-Flp-In 293 stable cells. hTRPA1-Flp-In 293 stable cells were successfully activated by a specific agonist for hTRPA1, AITC (30 μM) and five compounds. According to quantitative analysis, the effects of five active compounds on hTRPA1-Flp-In 293 stable cells were significantly inhibited by two hTRPA1 antagonists, RR (30 μM) and HC-030031 (100 μM). (B) The effects of the ten compounds from *A*. *rugose* were monitored in HEK293T cells transiently expressing hTRPV1 by Ca^2+^ imaging and counting the responding cells (%). hTRPV1 was significantly stimulated by capsaicin (0.1 μM), a specific agonist for hTRPV1, but not by ten compounds. Statistical analysis is specifically indicated for each experiment (**p<0.001, ***p<0.0001).

Of the ten tested compounds (acacetin, 4-allylanisole, *p*-anisaldehyde, apigenin 7-glucoside, *L*-carveol, *β*-caryophyllene, *trans*-*p*-methoxycinnamaldehyde, methyl eugenol, pachypodol, and rosmarinic acid), 4-allylanisole (1 mM), *p*-anisaldehyde (1 mM), *L*-carveol (300 μM), *trans*-*p*-methoxycinnamaldehyde (100 μM), and methyl eugenol (300 μM) activated hTRPA1 (Fig 3A). By quantitative analysis, the cytosolic Ca^2+^ influxes induced by 4-allylanisole (1 mM), *p*-anisaldehyde (1 mM), *L*-carveol (300 μM), *trans*-*p*-methoxycinnamaldehyde (100 μM), and methyl eugenol (300 μM) in hTRPA1-Flp-In 293 stable cells were mostly blocked by 30 μM RR and 100 μM HC-030031 (Fig 3). On the other hand, none of the compounds affected the intracellular [Ca^2+^] in non-hTRPA1-expressing Flp-In 293 cells (data not shown) and HEK293T cells transiently expressing hTRPV1 (Fig 3B).

**Fig 4:**
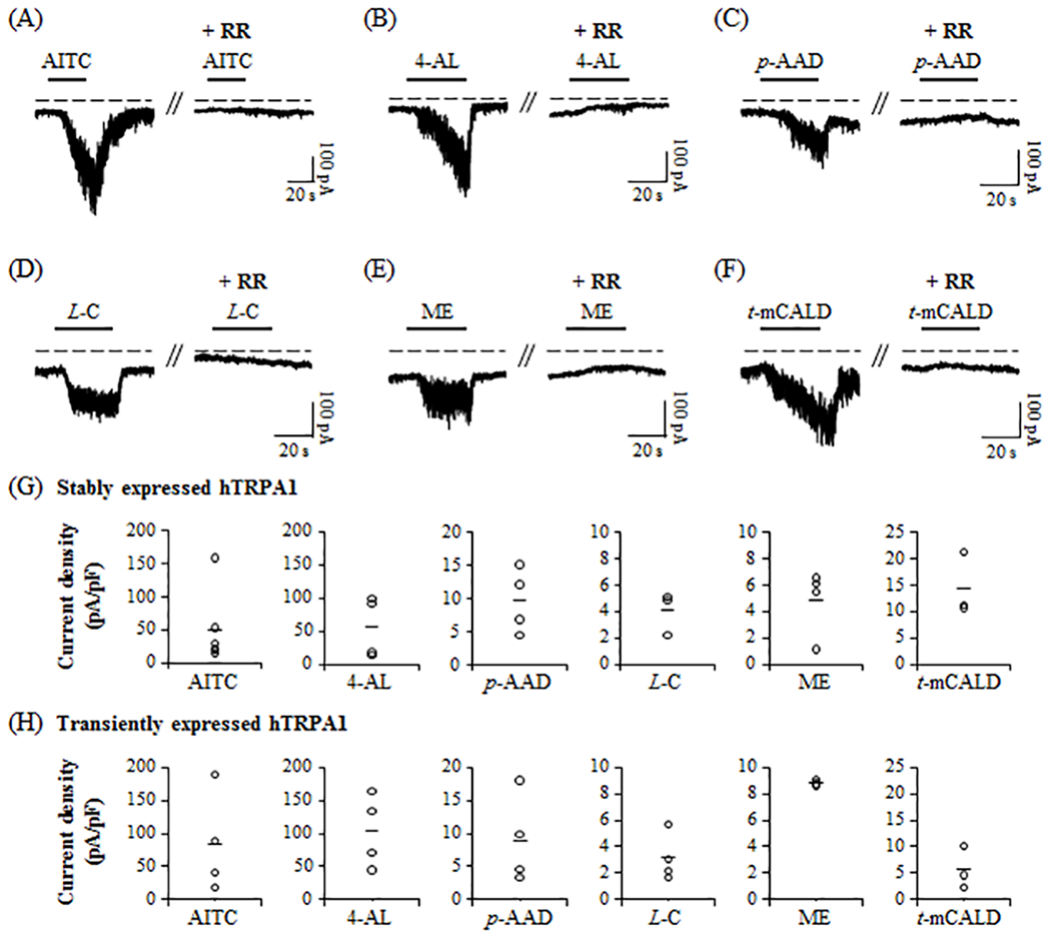
Activation of hTRPA1 currents by compounds. Application of (A) AITC (30 μM), (B) 4-allylanisole (3 mM), (C) *p*-anisaldehyde (6 mM), (D) *L*-carveol (1.8 mM), (E) methyl eugenol (900 μM), and (F) *trans*-*p*-methoxycinnamaldehyde (1 mM) for 30 s triggered RR-sensitive inward currents in hTRPA1-Flp-In 293 stable cells. The hTRPA1 currents activated by each compounds were completely blocked by pre-incubation of cells by extracellular solution containing RR (30 μM) for 30 s before the second application of compounds (n = 3 for AITC, n = 3 for 4-allylanisole, n = 4 for methyl eugenol, n = 4 for *p*-anisaldehyde, n = 3 for *L*-carveol, and n = 3 for *trans*-*p*-methoxycinnamaldehyde). The current density of hTRPA1 currents activated by each compounds (AITC (30 μM), 4-allylanisole (3 mM), *p*-anisaldehyde (6 mM), *L*-carveol (1.8 mM), methyl eugenol (900 μM), and *trans*-*p*-methoxycinnamaldehyde (1 mM) for 30 s) in hTRPA1-Flp-In 293 stable cells (G) and HEK293T cells transiently expressing hTRPA1 (H). The current density in y-axis shows the mean of each data.

To verify whether the compounds can activate hTRPA1, we patch-clamped in either hTRPA1-Flp-In 293 stable cells or HEK293T cells transiently expressing hTRPA1 (Fig 4). AITC (30 μM), 4-allylanisole (3 mM), *p*-anisaldehyde (6 mM), *L*-carveol (1.8 mM), methyl eugenol (900 μM), and *trans*-*p*-methoxycinnamaldehyde (1 mM) induced inward currents in both hTRPA1-expressing cells (Fig 4). The currents evoked by AITC and five compounds were completely blocked by pre-incubation of cells with extracellular solution containing 30 μM RR for 30 s before the second application of compounds (Fig 4). These results suggest that 4-allylanisole, *p*-anisaldehyde, *L*-carveol, methyl eugenol, and *trans*-*p*-methoxycinnamaldehyde can activate RR-sensitive hTRPA1 currents.

### Quantitative changes in hTRPA1-expressing cells treated with 4-allylanisole, *p*-anisaldehyde, *L*-carveol, *trans*-*p*-methoxycinnamaldehyde, and methyl eugenol

**Fig 5:**
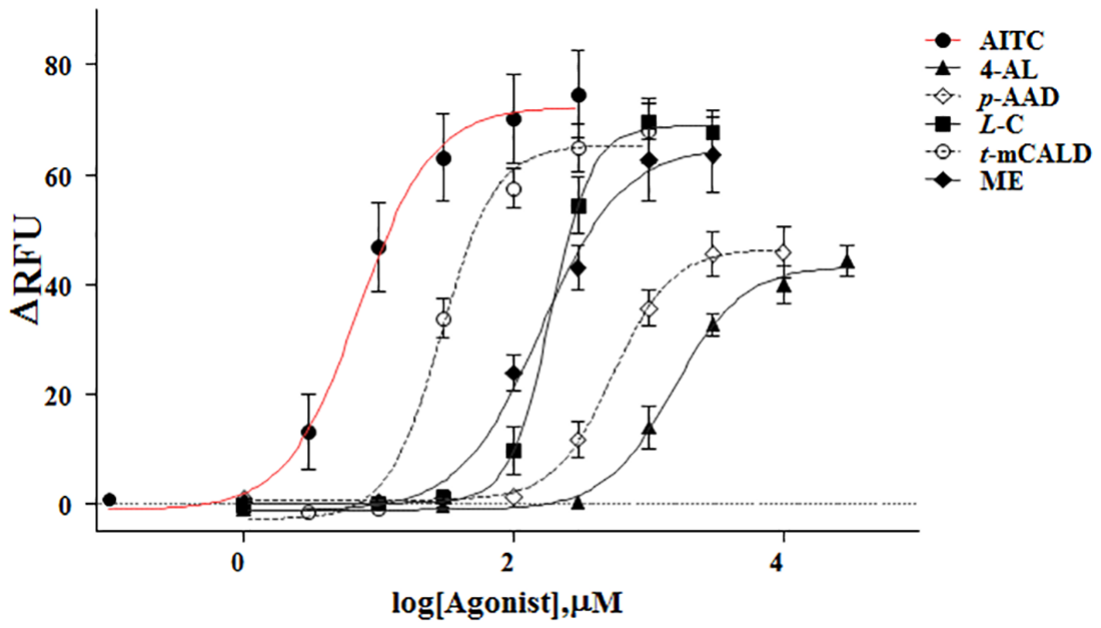
Effects of 4-allylanisole, *p*-anisaldehyde, *L*-carveol, *trans*-*p*-methoxycinnamaldehyde, and methyl eugenol on hTRPA1-Flp-In 293 stable cells. Data showed concentration-dependent responses of hTRPA1-Flp-In 293 stable cells to AITC, 4-allylanisole, *p*-anisaldehyde, *L*-carveol, *trans*-*p*-methoxycinnamaldehyde, and methyl eugenol. Each column shows the mean ± SEM (n = 7).

Fig 5 shows the dose-response curves for the Ca^2+^ responses induced by AITC, 4-allylanisole, *p*-anisaldehyde, *L*-carveol, *trans*-*p*-methoxycinnamaldehyde, and methyl eugenol in hTRPA1-Flp-In 293 stable cells. AITC and 4-allylanisole (1 μM to 30 mM), *p*-anisaldehyde (1 μM to 10 mM), *L*-carveol (1 μM to 3 mM), *trans*-*p*-methoxycinnamaldehyde (1 μM to 1 mM), and methyl eugenol (1 μM to 3 mM) produced concentration-dependent increases in the cytosolic [Ca^2+^] in hTRPA1-Flp-In 293 stable cells. All test compounds reached a plateau at their final concentrations. The EC_50_ value for each compound was calculated from concentration-response curves obtained from seven independent experiments. AITC had an EC_50_ value of 7.2±1.4 μM (Hill slope 1.6±0.5). The EC_50_ values for *L*-carveol, *trans*-*p*-methoxycinnamaldehyde, methyl eugenol, 4-allylanisole, and *p*-anisaldehyde were estimated as 189.1±26.8 (Hill slope 2.9±0.5), 29.8±14.9 (Hill slope 2.2±0.7), 160.2±21.9 (Hill slope 1.4±0.3), 1535.0±315.7 (Hill slope 1.7±0.3), and 546.5±73.0 μM (Hill slope 2.0±0.5), respectively. Among five compounds, 4-allylanisole and *p*-anisaldehyde were partial agonists with efficacies 59.9 and 64.2% that of AITC, respectively.

## Discussion

In the present study, we discovered that an extract of the stem and leaves of *A*.*rugosa* activated two nonselective chemosensory cation channels, hTRPA1 and hTRPV1 but ten commercially available compounds it contains primarily activated only hTRPA1. TRPA1 and TRPV1 are mediators of inflammation. Considering that *A*.*rugosa* inhibits inflammatory activity, the anti-inflammatory effects of *A*.*rugosa* can occur via hTRPA1- and hTRPV1-mediated pathways. In addition to anti-inflammatory activity, *A*.*rugosa* also has anti-fungal and anti-bacterial effects and it inhibits cytokine-induced vascular cell adhesion molecule-1 in human umbilical vein endothelial cells [16,17] and apoptosis in leukemia cells [23]. Of the ten chemical compounds in *A*.*rugosa*, *trans*-*p*-methoxycinnamaldehyde, *L*-carveol, methyl eugenol, *p*-anisaldehyde, and 4-allylanisole activated hTRPA1 with EC_50_ values of 29.8±14.9, 189.1±26.8, 160.2±21.9, 546.5±73.0, and 1535±315.7 μM, respectively, but did not activate hTRPV1. These values are reliable because the EC_50_ value for AITC in this study (7.2 μM) was similar to those in the literature (3–34 μM) [4,10]. Although the EC_50_ values for the five compounds were higher than that for AITC, they were specific agonist of hTRPA1 whose effects were blocked by HC-030031. In addition, *trans*-*p*-methoxycinnamaldehyde, *L*-carveol, and methyl eugenol were almost full agonists, while *p*-anisaldehyde and 4-allylanisole were potent partial agonists at hTRPA1 because the maximum ΔRFU of 4-allylanisole and *p*-anisaldehyde were 59.9 and 64.2% that of AITC, respectively. Some partial agonists can reduce intrinsic activity compared to a full agonist. Considering EC_50_ and the maximum ΔRFU induced by each compound, *trans*-*p*-methoxycinnamaldehyde showed the highest potency and efficacy among ten chemical compounds.

TRPA1 is activated by several mechanisms including non-covalent or covalent modification and secondary products. However, the predominant mechanism to activate TRPA1 is direct covalent modification. Many TRPA1 agonists are electrophiles in biological environment and chemically react with cysteine residues, nucleophiles, in TRPA1 by a Michael’s addition [3,24]. AITC structurally containing alkene and isothiocyanate is also electrophiles, so binds to cysteine residue. A triple TRPA1 cysteine mutant (C619/C639/C663) in N-terminal is presumed to be the binding sites of AITC in TRPA1 because the response to AITC in the TRPA1 mutant was significantly reduced, compared to in the WT [24]. Because many isothiocyanate derivatives activate TRPA1, isothiocyanate was concerned as a binding site to cysteine residue. However, isothiocyanate is not directly reacted with cysteine and no other mechanism is known. There is one possibility that the covalent bond between alkene in AITC and a thiol in cysteine residue can be performed (R-CH = CH_2_ + HS-R' → R-CH_2_-CH_2_-S-R'). 4-allylanisole, *L*-carveol, and methyl eugenol also contain alkene group.

From this logic, it is possible that alkene group in 4-allylanisole, methyl eugenol, and *L*-carveol can covalently bind to cysteine residue in TRPA1. Methyl eugenol and 4-allylanisole are phenylpropenes [25] and their structures are related to that of eugenol (found in cloves). Even though eugenol and methyl eugenol only differ by one functional group (the alcohol group in eugenol is replaced by a methoxy group in methyl eugenol), eugenol activates TRPA1, TRPV1, and TRPV3 [26], but methyl eugenol activates TRPA1 but not TRPV1. Thus, the activity of methyl eugenol is not identical to that of eugenol. However, hTRPA1-mediated phenomena induced by methyl eugenol might be similar to those induced by eugenol. *L*-carveol is a monocyclic monoterpenoid alcohol that is structurally similar to limonene, a monoterpene. Found in citrus fruits, limonene is a TRPA1 agonist, but not a TRPV1 agonist. The activities of both *L*-carveol and limonene on TRPA1 and TRPV1 are identical, so their pharmacological effect may be similar to each other.

Of the five hTRPA1 activators, *trans*-*p*-methoxycinnamaldehyde showed the strongest activity towards hTRPA1 because of its structural similarity to CALD, a strong hTRPA1 agonist found in cinnamon oil. CALD activates hTRPA1 by covalently modifying cysteine residues in its N-terminus [27], especially TRPA1 cysteine mutant (C621S/C641S/C665S). CALD composed of unsaturated aldehyde is strong electrophiles, so β-carbon in CALD is reacted with thiol group at cysteine residue by Michael’s addition and covalent bond is formed (CHO-CH = CH_2_-R + HS-R' → CHO-CH_2_-CH_2_(R)-S-R'). *Trans*-*p*-methoxycinnamaldehyde also contains unsaturated aldehyde. Therefore, it is expected that *trans*-*p*-methoxycinnamaldehyde and CALD activate hTRPA1 with almost identical activities and efficacies. CALD has several biological effects, including anti-inflammatory effect, delayed gastric emptying and antimicrobial, anti-fungal, and anti-bacterial activities. CALD binds to hTRPA1 in enterochromaffin cells; causes the release of intestinal hormones such as cholecystokinin, glucagon-like peptide-1, and gastric inhibitory polypeptide; and delays gastric emptying [28]. In addition, CALD has antispasmodic, antidiarrheal, and antimicrobial pharmacological properties. *Trans*-*p*-methoxycinnamaldehyde may act in the same way as CALD and have similar effects. Furthermore, *trans*-*p*-methoxycinnamaldehyde suppressed human respiratory syncytial viral entrance in a human larynx carcinoma cell line [29].

Para-anisaldehyde is also composed of unsaturated aldehyde which expected to react with cysteine residue in TRPA1. An isomer of *p*-anisaldehyde, *o*-anisaldehyde activates TRPA1 through electrophilic modification. Although the position of its alcohol group is different, *p*-anisaldehyde was also able to activate hTRPA1.

In summary, this is the first study to evaluate the effects of an extract of *A*.*rugosa* and ten of its constituents on hTRPA1- and hTRPV1-expressing cells. Our results demonstrate that *trans*-*p-*methoxycinnamaldehyde, *L*-carveol, methyl eugenol, *p*-anisaldehyde, and 4-allylanisole activated hTRPA1-expressing cells by a TRPV1-independent mechanism. We suggest that *trans*-*p*-methoxycinnamaldehyde, *L*-carveol, methyl eugenol, *p*-anisaldehyde, and 4-allylanisole are new TRPA1 agonists. Human TRPA1 is abundantly expressed in sensory neurons and a number of organs, including the lungs, gastrointestinal organs, bladder, and visceral and vascular organs. The regulation of TRPA1 has physiological relevance in the human body, especially in the somatosensory system [30,31] and neurogenic inflammation [32] via sensory nerve activation. Furthermore, AITC and CALD delay gastric emptying, induce satiety, and control food intake [33]. Previously, we also reported that the TRPA1 agonist methyl syringate significantly suppressed food intake and delayed gastric emptying by elevating plasma PYY in male ICR mice [15]. Therefore, finding proper TRPA1-targeting molecules in *A*.*rugosa* may provide beneficial effects related to homeostasis as well as somatosensory properties and neurogenic inflammation.
